# Effect of very long-term storage and multiple freeze and thaw cycles on 11-dehydro-thromboxane-B_2_ and 8-iso-prostaglandin F_2α,_ levels in human urine samples by validated enzyme immunoassays

**DOI:** 10.1038/s41598-024-55720-3

**Published:** 2024-03-06

**Authors:** Giovanna Petrucci, Duaa Hatem, Ruth Langley, Siobhan Cleary, Aleksandra Gentry-Maharaj, Dario Pitocco, Alessandro Rizzi, Paola Ranalli, Francesco Zaccardi, Aida Habib, Bianca Rocca

**Affiliations:** 1 Department of Bioethics and Safety, Section of Pharmacology, Catholic University School of Medicine, Rome, Italy; 2 Fondazione Policlinico Universitario A. Gemelli IRCCS, Rome, Italy; 3 Medical Research Council (MRC) Clinical Trials Units at University College London (UCL), London, UK; 4 Diabetology Unit, Fondazione Policlinico Universitario A. Gemelli IRCCS, Rome, Italy; 5 Department of Hematology, S. Spirito Hospital, Pescara, Italy; 6 Leicester Real World Evidence Unit, Leicester Diabetes Centre, University of Leicester, Leicester, UK; 7 Department of Basic Medical Sciences, College of Medicine, QU Health, Qatar University, Doha, Qatar

**Keywords:** Biomarkers, Medical research

## Abstract

Biological samples are often frozen and stored for years and/or thawed multiple times, thus assessing their stability on long-term storage and repeated freeze–thaw cycles is crucial. The study aims were to assess:—the long-term stability of two major enzymatic and non-enzymatic metabolites of arachidonic acid, i.e. urinary 11-dehydro-thromboxane-(Tx) B_2_, 8-iso-prostaglandin (PG)F_2α_, and creatinine in frozen urine samples;—the effect of multiple freeze–thaw cycles. Seven-hundred and three urine samples measured in previously-published studies, stored at −40 °C, and measured for a second time for 11-dehydro-TxB_2_ (*n* = 677) and/or 8-iso-PGF_2α_ (*n* = 114) and/or creatinine (*n* = 610) were stable over 10 years and the 2 measurements were highly correlated (all rho = 0.99, *P* < 0.0001). Urine samples underwent 10 sequential freeze–thaw cycles, with and without the antioxidant 4-hydroxy-2,2,6,6-tetramethylpiperidin-1-oxyl (10 mM); urinary 11-dehydro-TxB_2_ and creatinine were stable across all cycles (11-dehydro-TxB_2_: 100.4 ± 21%; creatinine: 101 ± 7% of baseline at cycle ten; *n* = 17), while 8-iso-PGF_2α_ significantly increased by cycle 6 (151 ± 22% of baseline at cycle ten, *n* = 17, *P* < 0.05) together with hydrogen peroxide only in the absence of antioxidant. Arachidonic acid metabolites and creatinine appear stable in human urines stored at −40 °C over 10 years. Multiple freeze–thaw cycles increase urinary 8-iso-PGF_2α_ in urine samples without antioxidants. These data are relevant for studies using urine samples stored over long-term and/or undergoing multiple freezing–thawing.

Urine samples donated alongside large clinical cohorts are usually frozen and stored for years or even decades after collection. Therefore, assessing the long-term stability of specific metabolites is crucial for reliable measurements over time and data interpretation. Arachidonic acid (AA) is a polyunsaturated fatty acid, released from membrane phospholipids by phospholipase A_2_ (PLA_2_) and in humans undergoes enzymatic and non-enzymatic biotransformation^1,2^. In activated platelets, AA is enzymatically transformed mainly via cyclooxygenase (COX) and thromboxane (Tx) synthase into TxA_2_, that is short-lived, and non-enzymatically hydrated into the inactive, stable TXB_2_^2^. In the human liver, TxB_2_ is biotransformed by different enzymatic pathways into several stable final metabolites, excreted in urine (Fig. 1)^3^, with 11-dehydro-TxB_2_ being amongst the most abundant and largely generated by activated platelets^4–6^. Consistent with its origin, urinary 11-dehydro-TxB_2_ is enhanced in conditions at high atherothrombotic risk, e.g. diabetes mellitus, hypertension, acute coronary syndromes, and stroke^7,8^. Moreover, 11-dehydro-TxB_2_ has been shown in large prospective cohorts to be a biomarker predicting major future cardiovascular events including mortality, as well as non-cardiovascular mortality and cancer over 5–12 years follow-up^7–10^.

**Figure 1:**
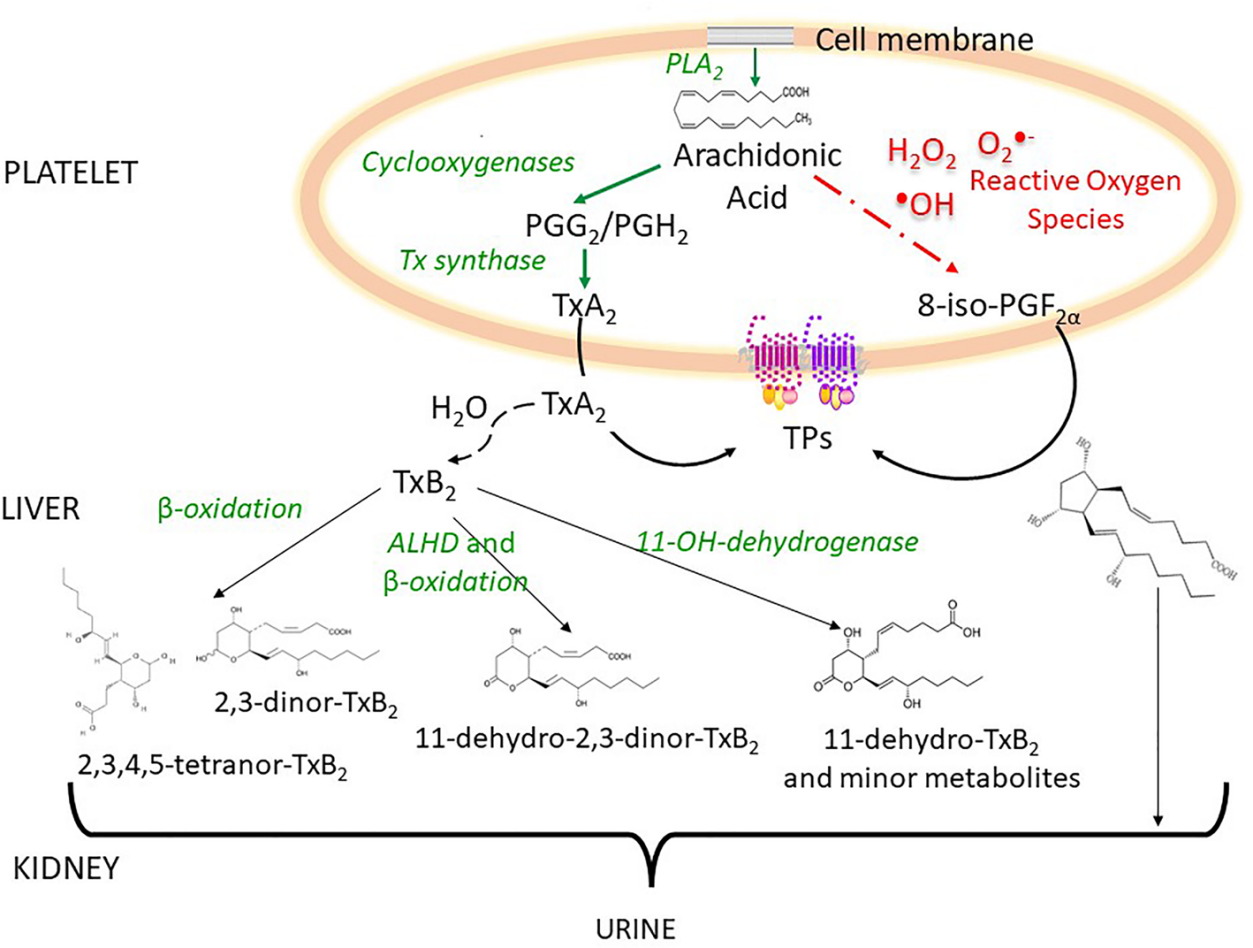
Metabolism of thromboxane A_2_/B_2_ and isoprostanes in humans. The figure shows the enzymatic and non-enzymatic metabolism of arachidonic acid toward thromboxane (Tx)A_2_/B_2_ and isoprostanes; approximately 70-80% of TxA_2_ generated daily in humans is released by platelets and rapidly hydrolysed to TxB_2_^3^ which is then enzymatically biotransformed in the liver into 20 stable metabolites, the main being 11-dehydro-TxB_2_ and 2,3-dinor-TxB_2_ excreted by the kidney. The non-enzymatic metabolism of arachidonic acid generates 8-iso-prostaglandin (PG)F_2α_ which is excreted unchanged in urine. ALHD: aldehyde dehydrogenase; PLA_2_: phospholipase A_2_; TPs: thromboxane A_2_ receptors.

By means of reactive oxygen species, AA is non-enzymatically oxidized into F_2_ isoprostanes that are excreted by the kidney (Fig. 1)^11^. In particular, the 8-iso-prostaglandin (PG)F_2α_ is the most abundant in human urine^12^, reflecting in vivo lipid peroxidation^2,13^. Consistently, urinary 8-iso-PGF_2α_ is increased in conditions at high cardiovascular risk as well as high oxidative stress such as cigarette smoking, diabetes mellitus hypercholesterolemia, and obesity^14^. In addition, urinary 8-iso-PGF_2α_ has been reported as an independent biomarker of future cardiovascular events and mortality^15,16^. In the large longitudinal Framingham cohort, urinary 8-iso-PGF_2α_ and 11-dehydro-TxB_2_ were significantly correlated^9^.

Thus, urinary 11-dehydro-TxB_2_ and 8-iso-PGF_2α_ metabolites have been measured in large longitudinal studies^8,9,17^, possibly years after collection, however their long-term stability has never been assessed while is rather relevant. Both metabolites contain cycloalkanes, double bonds, as well as oxygen and hydroxyl groups (Fig. 1)^18^, which may affect their long-term stability and antigenic properties in immunometric measurements.

Thus, the aims of this study were: (i) to investigate the effect of long-term storage on the concentration of 11-dehydro-TXB_2_, 8-iso-PGF_2α_ and creatinine (as a control molecule), in urine and chromatographic extracts of urine, all stored at −40 °C, and (ii) to assess the effect of repeated freeze–thaw cycles in urine samples and in chromatographic extracts.

## Materials and methods

### Study samples

Urine samples from 703 subjects (51 healthy individuals^19,20^, 61 patients with diabetes mellitus^20,21^[Petrucci et al. accepted for publication], 242 patients with hematologic^22,23^ and 349 with solid cancers^10^ were assayed a first time in previously-published studies^10,19–23^[Petrucci et al. accepted for publication]. In the original protocols, all urine samples were collected from study participants and frozen within 2–3 h from collection at −40 °C, under controlled temperature (PDF 440W, EVERmed, Medical Refrigeration; Motteggiana, MN and KBPF600 PP, KW Apparecchi Scientifici srl, Monteriggioni, SI, all in Italy) until first measurement. After the first measurement samples were stored at −40 °C, under controlled temperature until the present study.

For this study, urine samples were selected if: 1- there was a first measure of 11-dehydro-TxB_2_, creatinine and/or 8-iso-PGF_2α_, depending on the original protocol; 2- the remaining volume was sufficient for a second extraction and/or creatinine measure, i.e. ≥ 2 mL;—they were correctly stored under controlled temperature of -40 °C over the indicated time interval between 1 week and 10 years.

### Eleven-dehydro-TxB_2_ and 8-iso-PGF_2α_ measurements

Thawed samples were centrifuged at 671 g for 5 min (Centrifuge-5702, Eppendorf, Milan, Italy), 1 mL of the supernatant underwent chromatographic extraction, as previously described^24^. Briefly, 2000 cpm of ^3^H-PGE_2_ (3.70–6.86 TBq/mmol, Perkin Elmer, Boston, MA, USA) was added to 1 mL urine and loaded into a 1 mL/50 mg C18 column (Bakerbond™-SPE, J.T. Baker, Gliwice, Poland) and eluted with 2.5 mL isooctane/ethyl acetate (1:1, vol/vol). The first eluate was loaded into a 1 mL/100 mg SiOH column (Bakerbond™-SPE) and eluted with 2 mL ethyl acetate/methanol (60:40, vol/vol), samples were then dried and resuspended in 1 mL PBS/0.1% BSA buffer (pH 7.4) for immunoassay and assessment of recovery calculated on the ^3^H-PGE_2_ counting. The variability of the urine extraction method calculated on repeated extractions of the same samples was 12% over the entire study duration (*n* = 24 samples extracted multiple times).

For 11-dehydro-TxB_2_, 677 suitable urine samples, extracted as described above, were assayed by a standard enzyme-linked immunosorbent assay (ELISA) as previously published^25^, using a specific rabbit polyclonal antiserum^26^ with a detection range from 3.9 to 500 pg/mL and a sensitivity calculated as 80% B/B_0_ (i.e. the relative maximum binding in a sample to maximum binding capacity) of 10 pg/mL. The inter-assay variability, calculated as the coefficient of variation of repeated measurement of a commercial standard (11-dehydro-TxB_2_ ELISA Standard, Cayman Chemical, Ann Arbor, MI, USA), was 9% (*n* = 1344 determinations) over the entire study duration. The accuracy of the ELISA was assessed using a commercial standard of 1.5 ng/mL (Cayman Chemicals) that measured 1.57 ± 0.14 ng/mL (n = 20 measurements). The cross-reactivity of the anti-11-dehydro TxB_2_ antiserum against other prostanoids that can be measured in urine, namely PGE_2_ 2,3-dinor T_X_B_2_, TXB_2_, 6-keto PGF_1α_, and the isoprostane 8-iso-PGF_2α_ was < 0.05%, and against PGD_2_ was 0.3%.

For 8-iso-PGF_2α_, 114 suitable urine samples were processed and immuno-assayed as previously described^25^ with a specific rabbit polyclonal antiserum^27^ with a detection range from 3.9 to 500 pg/mL, the sensitivity of 9 pg/mL and an inter-assay coefficient of variation using a commercial standard (8-isoprostane ELISA Standard, Cayman Chemical) of 7.8% over the entire study duration (*n* = 194 determinations). The cross-reactivity for the anti-8-iso-PGF_2α_ antiserum against other urinary prostanoids was < 0.5% namely PGE_2,_ 2,3-dinor T_X_B_2_, TXB_2_, 2,3-dinor-6-keto PGF_1α_ was < 0.05%, and against PGD_2_ and 6-keto PGF_2α_ was 0.16%. The accuracy assessed with a certified commercial standard (Cayman Chemical) of 10 ng/mL measured 11.4 ± 1.3 ng/mL (*n* = 10 measurements).

The stability of 11-dehydro TxB_2_ and 8-iso-PGF_2α_ as also assessed in chromatographic extracts stored in PBS at −40 °C between 1 week and 10 years. Extracts were assayed again by ELISA for 11-dehydro TxB_2_ (*n* = 748 samples) and/or for 8-iso-PGF_2α_ (*n* = 212 samples) as described.

Both ELISA methods used in the current study had been previously validated against gas chromatography/mass-spectrometry (GC/MS) and showed a strong correlation between the analysis of identical urine samples for 11-dehydro TxB_2_ and 8-iso-PGF_2α_^25,27^.

### Creatinine measurements

For creatinine, 610 urine samples from 53 healthy individuals^19,20^, 189 patients with diabetes mellitus [Petrucci et al. submitted], 110 patients with hematologic^21,22^, and 258 with solid cancers^10^, were assayed for a second time for creatinine using a commercial kit (Creatinine Colorimetric Detection Kit; Enzo Life Sciences, Farmingdale, NY, USA). The inter-assay coefficient of variation using a commercial standard (Creatinine Standard, Cayman Chemical) was 7% over the study duration (n = 523 determinations).

### Freeze–thaw experiments

Forty urine samples from 37 volunteers were collected and aliquoted into 1.5 mL samples without antioxidant. One aliquot was immediately processed (baseline sample) and the remaining aliquots that were frozen at −80 °C for 20 min and thawed in water bath at 25 °C for 10 min multiple times. In 24 samples the antioxidant 4-hydroxy-2,2,6,6-tetramethylpiperidin-1-oxyl (4-hydroxy-TEMPO) (Merck KGaA, Darmstadt, Germany, 10 mM final concentration), was added immediately after collection and samples underwent multiple freeze thaw cycles as already described.

The urinary pH was measured at thawing cycles 2, 4, 8 and 10 using a pH tester (pH510, Eutech Instruments Europe B.V.—Landsmeer, The Netherlands).

To evaluate the oxidative stress we measured hydrogen peroxide (H_2_O_2_) levels, an established index of reactive oxygen species generation^28^,with a commercial kit (Peroxide Assay Kit, Merck KGaA) that measures H_2_O_2_ using a chromogenic Fe^3+^  − xylenol orange reaction based on the Fenton reaction. The colorimetric intensity is proportional to the H_2_O_2_ concentration in the sample.

The effect of freeze–thaw cycles was also investigated in the chromatographic extracted samples in PBS, by pooling extracts and making aliquots for multiple freeze–thaw cycles, as described.

### Statistical analysis

Since the second measurement was dependent on both the availability of a first measurement performed for a specific protocol as well as on an appropriate remaining sample volume, we did not formulate a specific hypothesis for sample size over time.

Data from the repeated measurements and the freeze–thaw experiments were plotted as % of the first or baseline measurement, respectively, as indicated. Data were expressed median and interquartile ranges. Comparisons were performed by ANOVA for repeated measurements or paired *t*-test, as appropriate. Correlations were analysed by Pearson or Spearman's rank test according to data distribution. The significance was set at *P* < 0.05. Analyses were performed using GraphPad Prism 7.04.

All samples were received anonymized in the central laboratory at the Catholic University School of Medicine in Rome (to GP and BR), since were labelled only using alphanumeric codes, according to the original protocols. Each study protocol was approved by the referent Ethics Committee, namely: South Central-Oxford C, UK, ref 14/SC/0171^10^, Rome, Italy, ref P/852/CE/2012^19^, ref. P/464/CE/2010^20^, ref 32(A.1668)/CE/2009, Center of Rome^21^, ref 28,371/16, ID 1285^23^*;* Santo Spirito Hospital, Pescara, Italy, ref 204 CE/2010^22^*;* North West Multi-centre Research Ethics Committee,0.29/12/2003, ref: 03/8/087, sub-study TXM [Petrucci et al. accepted for publication]. All protocols were performed in accordance with the Helsinki declaration^29^ and written informed consent for study participation and measurements of urinary metabolites was obtained from all study participants.

## Results

### Eleven-dehydro-TxB_2_, 8-iso-PGF_2α_ and creatinine in stored samples

#### Biomarkers in urine samples

**Figure 2:**
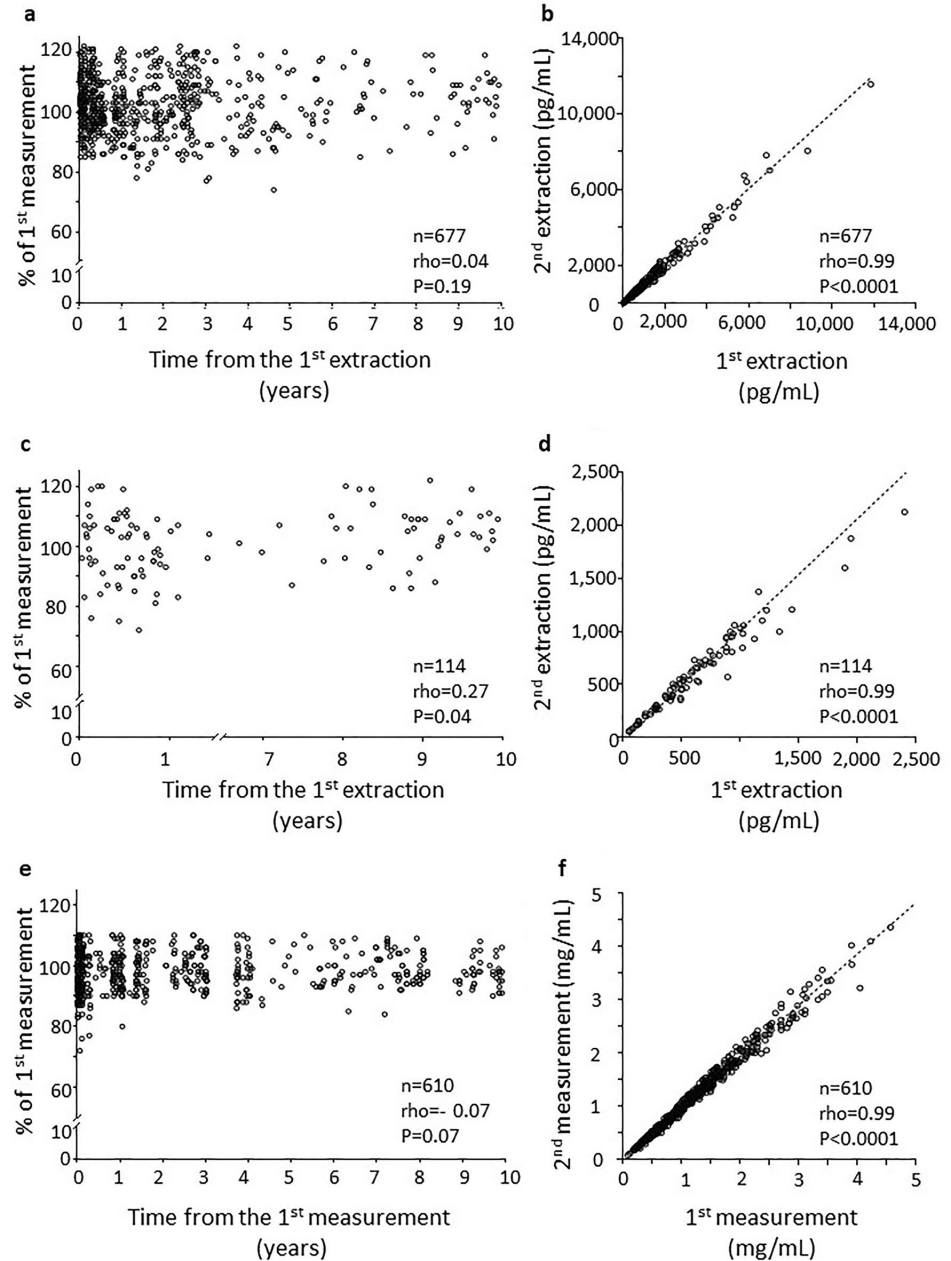
Eleven-dehydro-TxB_2_, 8-iso-PGF_2α_, and creatinine in urine samples stored for up to 10 years. Panel (**a**) shows 11-dehydro-TxB_2_ (pg/mL) values in urine samples stored from 1 week up to 10 years. Values are represented as % of the first extraction. Panel (**b**) represents the absolute values of 11-dehydro-TxB_2_ (pg/mL) on the first versus the second extraction. Dotted line is the correlation. Panel (**c**) shows 8-iso-PGF_2α_ (pg/mL) values in urine samples stored from 2 weeks up to 10 years. Values are % of the first extraction. Panel (**d**) represents the absolute values of 8-iso-PGF_2α_ (pg/mL) on the first versus the second extraction. Dotted line is the correlation. Panel (**e**) shows urinary creatinine values in urine samples stored from 1 week up to 10 years. Values are % of the first creatinine measurement. Panel (**f**) represents the absolute values of creatinine (mg/mL) on the first versus the second measurement. Dotted line is the correlation. PG: prostaglandin; Tx: thromboxane.

Eleven-dehydro-TxB_2_ levels, expressed as % of the first measurement, were stable in urine samples stored between 1 week and 10 years (*n* = 677, Fig. 2a). Values of the first and second measurement were similar (750.5 [348.2–11850] and 743.1 [354–11563] pg/mL, median and [interquartile range], respectively) and highly correlated (rho = 0.99, *P* < 0.0001, *n* = 677, Fig. 2b). Similarly, 8-iso-PGF_2α_ levels were largely stable between 2 weeks and 10 years (*n* = 114, Fig. 2c); overall, the second measurements were similar to the first ones (497 [279.6–850] and 507.6 [277.1–807.3] pg/mL, respectively) and highly correlated (rho = 0.99, *P* < 0.0001, *n* = 114, Fig. 2d).

We measured urinary creatinine as a reference molecule and based on the consideration that final 11-dehydro TxB_2_ and 8-iso-PGF_2α_ concentrations are corrected for creatinine excretion to adjust for kidney function (final values expressed as pg/mg creatinine). Creatinine concentrations were also stable (*n* = 610, Fig. 2e), reproducible (1.0 [0.55–1.54] and 0.99 [0.54–1.54] mg/mL, first and second measurement, respectively) and highly correlated (rho = 0.99, *P* < 0.0001, *n* = 610, Fig. 2f).

**Figure 3:**
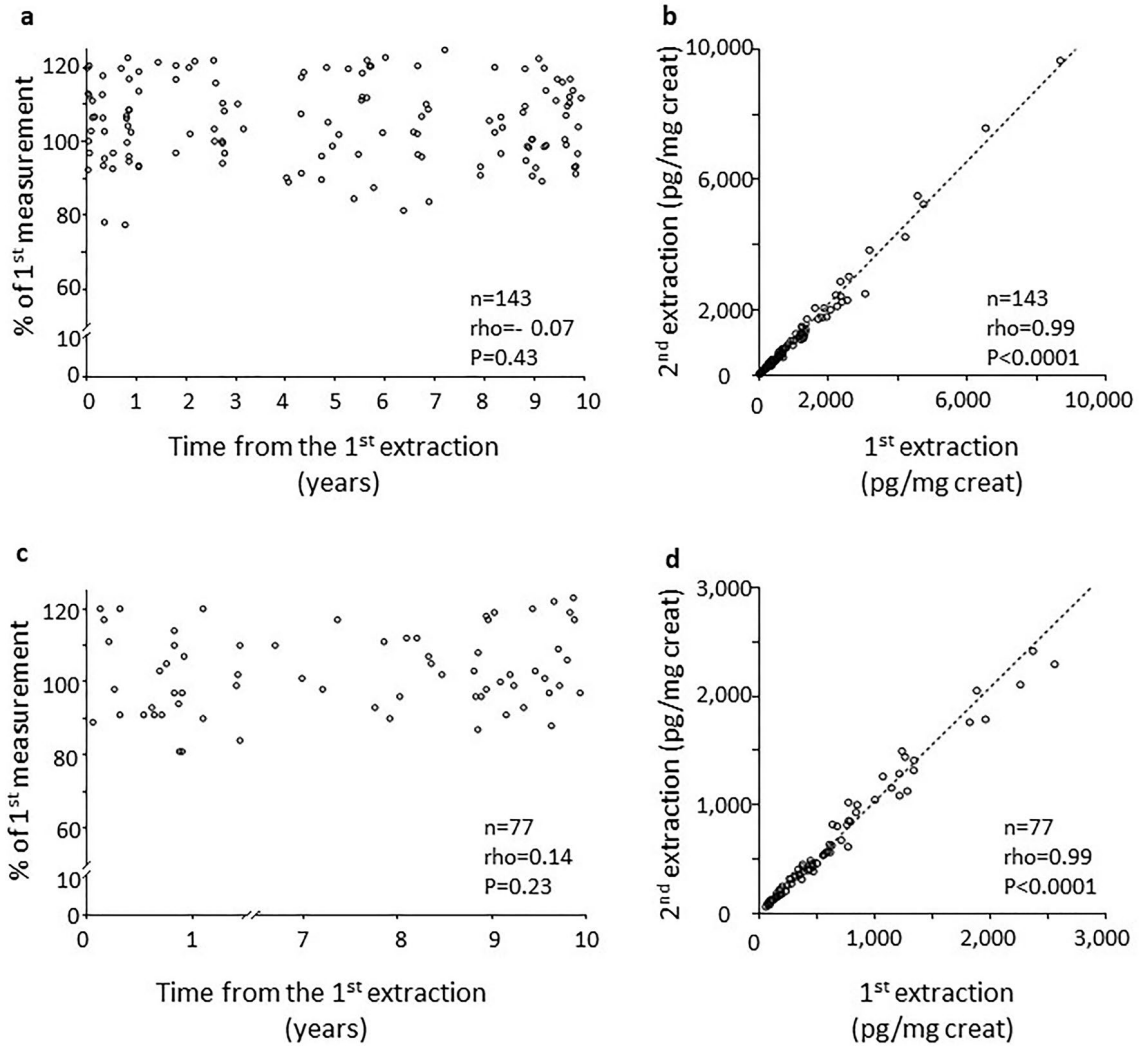
Eleven-dehydro-TxB_2_ and 8-iso-PGF_2α_ expressed as pg/mg creatinine in urine samples stored for up to 10 years. Panel (**a**) shows 11-dehydro-TxB_2_ (pg/mg creatinine) values in urine samples stored from 1 week up to 10 years. Values are % of the first extraction. Panel (**b**) represents the absolute values of 11-dehydro-TxB_2_ (pg/mg creatinine) on the first versus the second extraction. Dotted line is the correlation. Panel (**c**) shows urinary 8-iso-PGF_2α_ (pg/mg creatinine) values in urine samples stored from 2 week up to 10 years. Values are % of the first extraction. Panel (**d**) represents the absolute values of 8-iso-PGF_2α_ (pg/mg creatinine) on the first versus the second extraction. Dotted line is the correlation. PG: prostaglandin; Tx: thromboxane.

Based on volume availability, we measured 11-dehydro TxB_2_, 8-iso-PGF_2α_, as well as creatinine in some samples; 11-dehydro-TXB_2_ values expressed as pg/mg creatinine (*n* = 143), were also stable over 10-year storage (Fig. 3a), with similar concentrations (394.7 [185.7–1006] and 420 [201.6–1045] pg/mg creatinine, first and second determination, respectively) and highly correlated (rho = 0.99, *P* < 0.0001, *n* = 143, Fig. 3b). In 77 urine samples 8-iso-PGF_2α_ concentrations expressed as pg/mg creatinine did not significantly change over 10 years (Fig. 3c) with similar levels (470.2 [218.8–929.7] and 455 [234.5–1032] pg/mg creatinine, in the first and second measurement, respectively) and highly correlated (rho = 0.99, *P* < 0.0001, Fig. 3d).

#### Chromatographic extracted samples from urine

**Figure 4:**
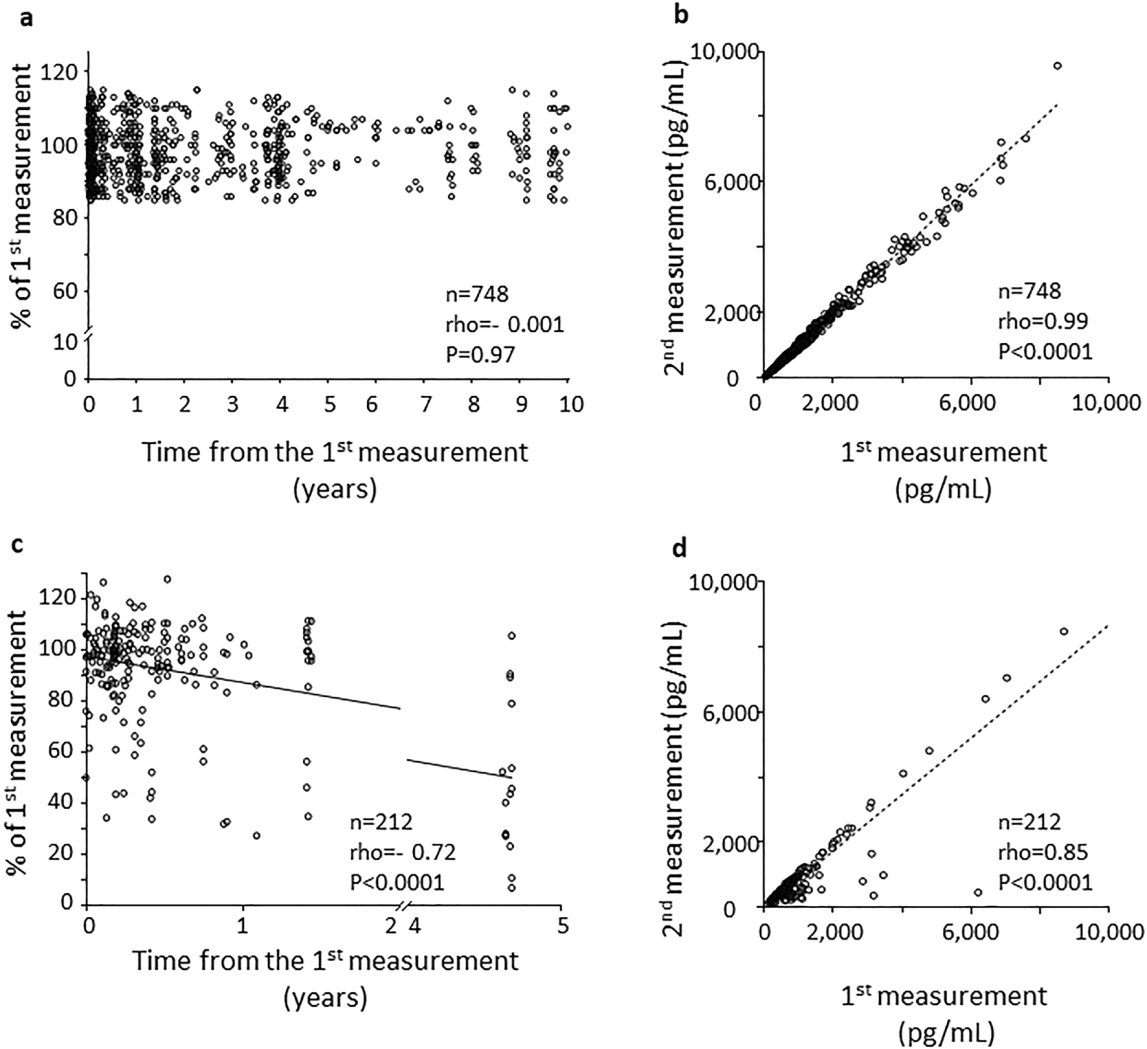
Eleven-dehydro-TxB_2_ and 8-iso-PGF_2α_ levels in chromatographic extracts stored for up to 10 years. Panel (**a**) shows 11-dehydro-TxB_2_ (pg/mL) values in chromatographic extracts stored in PBS from 1 week up to 10 years. Values are % of the first measurement. Panel (**b**) represents 11-dehydro-TxB_2_ (pg/mL) absolute values on the first versus the second measurement. Dotted line is the correlation. Panel (**c**) shows 8-iso-PGF_2α_ (pg/mL) repeated values in chromatographic extracts stored in PBS from 2 week up to 10 years. Values are % of the first measurement. Panel (**d**) represents 8-iso-PGF_2α_ (pg/mL) absolute values on the first versus the second measurement. Dotted line is the correlation. PG: prostaglandin; Tx: thromboxane.

As a control for the medium for the considered metabolites, we also performed immunoassays in chromatographic extracts of urine samples which were eluted and in PBS. In these extracts (*n* = 748), only the 11-dehydro-TXB_2_ was stable over time (Fig. 4a) with similar concentrations (543[210–1172] and 552.5[199–1181] mg/mL, first and second determination, respectively), with highly correlated values (rho = 0.99, *P* < 0.0001, Fig. 4b), while 8-iso-PGF_2α_ in chromatographic frozen extracts (*n* = 212) showed a significant trend toward a decrease starting approximately from month 4 of storage (Fig. 4c), even though the values of the 2 measurements were still significantly correlated (rho = 0.85, *P* < 0.0001, *n* = 212, Fig. 4d).

### Freeze–thaw cycles

**Figure 5:**
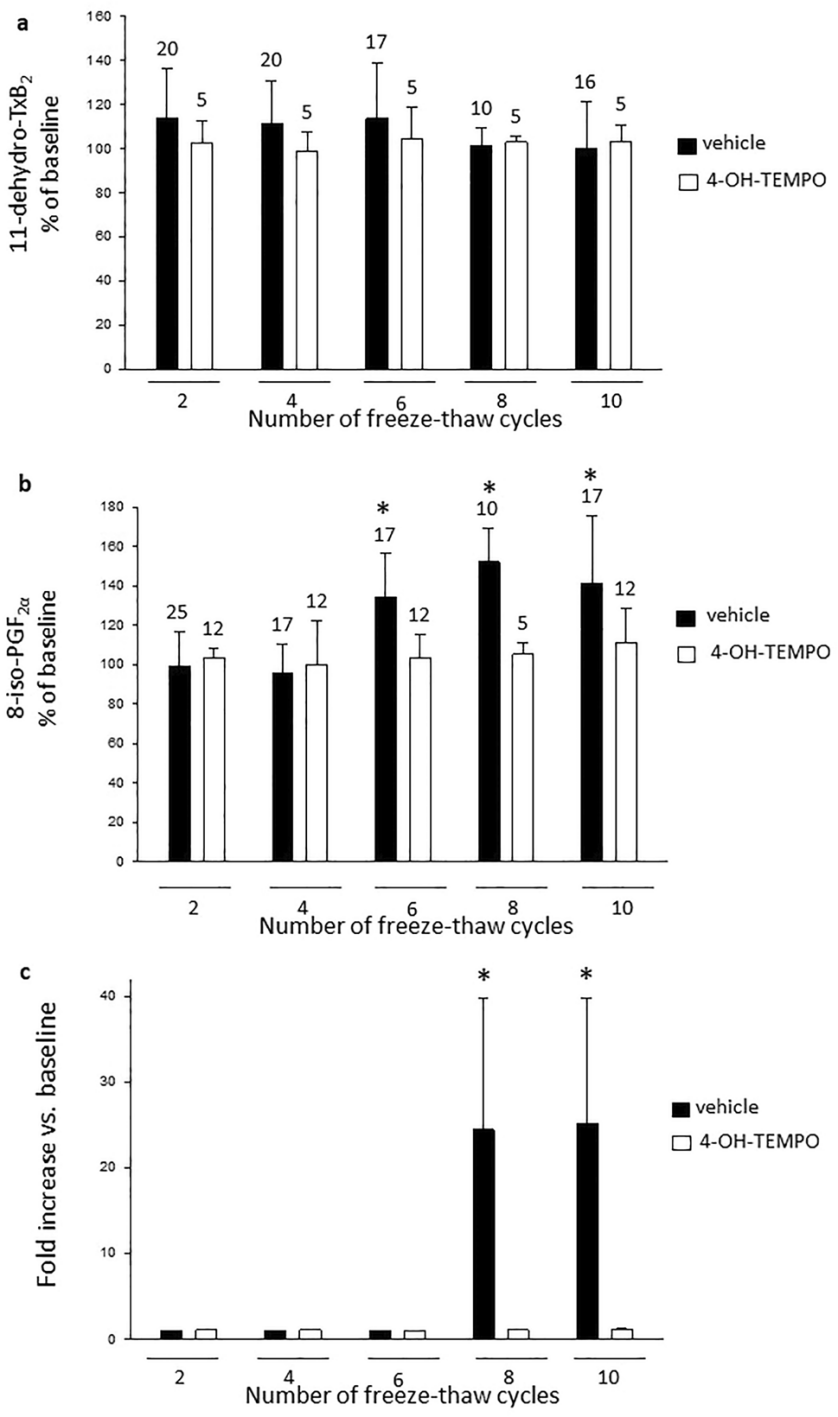
Effect of freeze-thawing cycles on 11-dehydro-TxB_2,_ 8-iso-PGF_2α_ and H_2_O_2_ in urine samples. Panel (**a**) shows the effect of up to 10 freeze-thaw cycles on 11-dehydro-TxB_2_ in urine samples with or without 10 Mm 4-OH-TEMPO. Panel (**b**) shows the effect of up to 10 freeze-thaw cycles on 8-iso-PGF_2α_ in urine samples with and without 10 mmol 4-OH-TEMPO. Panel (**c**) shows the effect of up to 10 freeze-thaw cycles on peroxides in urine samples (*n* = 3) with and without 10 mM 4-OH-TEMPO. Data are expressed as percentage of the corresponding baseline values and are means ± SD. *P < 0.0001 versus baseline. 4-OH-TEMPO: 4-hydroxy-2,2,6,6-tetramethylpiperidin-1-oxyl; PG; prostaglandin; Tx: thromboxane.

**Figure 6:**
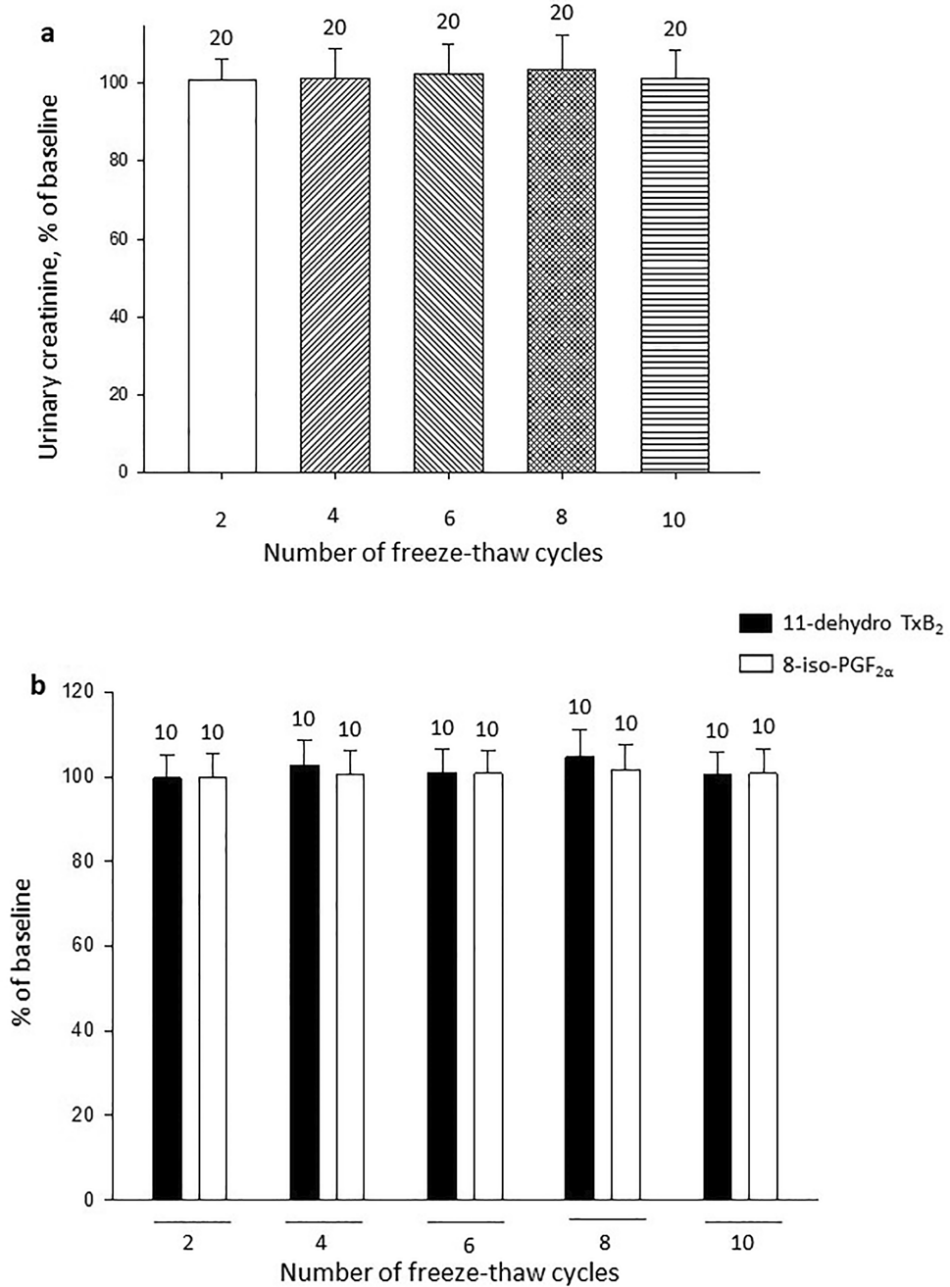
Effect of freeze–thaw cycles on urinary creatinine and on 11-dehydro-TxB_2,_ 8-iso-PGF_2α_ in chromatographic-extracts. Panel (**a**) shows the effect of 10 freeze-thaw cycles on creatinine in urine samples, each column represents means ± SD of *n* = 20 measurements. Panel (**b**) shows the effect of 10 freeze-thaw cycles on 11-dehydro-TxB_2_ and 8-iso-PGF_2α_ in chromatographic-extracted samples, each column represents means ± standard deviations of *n* = 10 determinations. Data are expressed as percentage of the corresponding baseline values. All values are not significantly different versus baseline. 4-OH-TEMPO: 4-hydroxy-2,2,6,6-tetramethylpiperidin-1-oxyl; PG; prostaglandin; Tx: thromboxane.

In urine samples without added antioxidant, 11-dehydro-TxB_2_ and creatinine concentrations were stable over 10 freeze–thaw cycles and were 100.4 ± 21% (*n* = 17) and 101 ± 7% of baseline (*n* = 20), respectively at cycle 10 (Fig. 5a and 6a). However, 8-iso-PGF_2α_ concentrations showed a significant increase starting from cycle 6, being 134 ± 9% of baseline at cycle 6 (*n* = 17, *P* < 0.001, Fig. 5b). We also measured H_2_O_2_ concentration in urine to assess whether oxidation products were increased by multiple freezing and thawing, and observed a parallel, significant 24.4 ± 15-fold increase vs. baseline by cycle 8 (*P* < 0.0001, Fig. 5c). When the antioxidant was added to urine samples, the urinary 8-iso-PGF_2α_ and H_2_O_2_ levels remained stable over the 10 freeze–thaw cycles (Fig. 5b,c).

At variance with urine samples, 11-dehydro TxB_2_ and 8-iso-PGF_2α_ in the chromatographic extracts were stable over the 10 freeze–thaw cycles (Fig. 6b).

The urinary pH values with and without antioxidant were not affected by the 10 freeze–thaw cycles (data not shown).

## Discussion

This study investigated for the first time the stability of the 2 major urinary enzymatic and non-enzymatic metabolites of AA, i.e., the 11-dehydro-TxB_2_ and the 8-iso-PGF_2α_, respectively, as well as of creatinine as a control molecule, in a large number of urine samples stored at –40º C for several years and in chromatographic urinary extracts, as control for the storage medium for these analytes (urine versus PBS in purified extracts). We also assessed the effect of multiple freeze–thaw cycles on the same metabolites in both urine and extract samples. To the best of our knowledge, this study has the largest sample size and the longest storage interval assessing stability.

In over 700 urine samples, we observed a substantial stability of 11-dehydro-TxB_2_, 8-iso-PGF_2α_, and creatinine levels between few weeks and 10 years, with a variability of the repeated values that remained within the coefficient of variation of the methods. We included different type of subjects from different studies, to evaluate whether in protein- and glucose-enriched urine, as in the case of diabetes mellitus, there were differences in the stability of the studied metabolites and creatinine. The stability of 11-dehydro-TxB_2_ and 8-iso-PGF_2α_ in urine samples stored at −40 °C over 10 years, was observed in all studied groups, i.e. healthy, diabetes mellitus and cancer subjects (Table 1). Previous studies on the 8-iso-PGF_2α_ reported stability in urine stored at −20 °C for up to 6 months^30^, at −70 °C for a maximum of 2 years (Table 2)^31^, and a recent study showed that both 11-dehydro-TxB_2_ and 8-iso-PGF_2α_ are stable in urine samples stored at both −20 and −70 °C over 3 years (Table 2)^32^. Thus, our data are consistent with and enlarge evidence from previous, smaller studies. Notably, our urine samples were stored at −40 °C for 10 years, which is a condition more feasible and cheaper as compared to lower storage temperatures, e.g. −80 °C, especially in large biobanks.

**Table 1:** Concentrations of 11-dehydro-TXB_2_, 8-iso PGF_2α_, and creatinine in urine samples stored for up to 10 years, overall and in each subgroup.

Groups	11-dehydro-TXB_2_ (pg/mL)			8-iso-PGF_2α_ (pg/mL)			Creatinine (mg/mL)		
	*n*	Time (years) vs % of the 1st extraction (rho)	1st vs 2nd measurement (rho)	n	Time (years) vs % of the 1st extraction (rho)	1st vs 2nd Measurement (rho)	*n*	Time (years) vs % of the 1st extraction (rho)	1st vs 2nd Measurement (rho)
All samples^10,19–22^	677	0.04 *P* = 0.3	0.99 *P* < 0.0001	114	0.27 *P* = 0.04	0.99 *P* < 0.0001	610	−0.07 *P* = 0.83	0.99 *P* < 0.0001
Healthy subjects^19,20^	51	0.24 *P* = 0.1	0.99 *P* < 0.0001	55	0.17 *P* = 0.2	0.99 *P* < 0.0001	53	0.09 *P* = 0.5	0.99 *P* < 0.0001
Diabetic patients^20,21^	35	0.11 *P* = 0.5	0.99 *P* < 0.0001	59	−0.12 *P* = 0.4	0.99 *P* < 0.0001	189	0.02 *P* = 0.8	0.99 *P* < 0.0001
Cancer patients^10,22,23^	591	0.03 *P* = 0.5	0.99 *P* < 0.0001	NA	NA	NA	368	0.12 *P* = 0.04	0.99 *P* < 0.0001

PG; prostaglandin; NA: data not available; TX: thromboxane.

**Table 2:** Effect of storage conditions and freeze–thaw cycles on different compounds in urine samples.

*Storage conditions*				
Study (year)	Study design	Storage conditions	Study population	Results
Spierto et al.^35^	Stability of urinary creatinine under different storage conditions	30 days at 4, 25, 37, and 55 °C	Healthy individuals (*n* = 10)	Creatinine levels remained stable at 4, 25, 37 °C, but significantly decreased at 55 °C as compared to the baseline;—42%; *P* < 0.05
Pagliaccia et al.^24^	Stability of 11-dehydro-TxB_2_, 8-iso-PGF_2α_ and creatinine in urine samples under different storage conditions	7 days at 4 and 25°C with and without antioxidant (1mM 4-OH-TEMPO)	Healthy individuals (*n* = 11) and patients with type 2 diabetes mellitus (*n* = 15)	All urinary analytes remained stable in urine samples
Remer et al.^37^	Long-term stability of Creatinine, urea, chloride, phosphate, sodium, potassium, calcium, magnesium, citrate, uric acid, iodine, and nitrogen in urine samples from the Dortmund Nutritional and Anthropometric Longitudinally Designed Study	12–15 years at −22 °C	Healthy children (*n* = 10)	All urinary analytes were stable over 15 years
Holder et al.^31^	Stability of 8-iso-PGF_2α_ while developing an analytical method by mass spectrometry	2 years at −70 °C	2 urine pools from healthy subjects	8-iso-PGF_2α_ values were stable over 2 years
Sieminska et al.^32^	Stability of 11 dehydro-TxB_2,_ 8-iso-PGF_2α_ and other 17 eicosanoids while developing an analytical method by a single extraction 96-well method for LC–MS/MS quantification	3 years at −20 and −70 °C	5 urine pools from healthy subjects	Polar tetranors (tetranor-PGDM, -PGFM, -PGEM and -PGAM) showed a 50% of reduction after 5 months at −20°C, while 11 dehydro-TxB_2,_ 8-iso-PGF_2α_ and other eicosanoids were stable over 3 years at −20°C All urinary analytes were stable over 3 years at −70°C
Present study	Stability of 11-dehydro-TxB_2_, 8-iso-PGF_2α_ and creatinine in frozen urine samples and in chromatographic extracts of urine over a long time interval	To 1 week up to 10 years at −70 °C	Healthy individuals (*n* = 51), patients with diabetes (*n* = 61), patients with hematologic (*n* = 242) and solid cancers (*n* = 349)	11-dehydro-TxB_2_, and creatinine were stable in urine samples and in their chromatographic extracts up to 10 years at −40 °C 8-iso-PGF_2α_ was stable in urine samples up to 10 years at −40 °C, but significantly decreased in chromatographic extracts of urine starting approximately from month 4 of storage; rho = −0.72; P < 0.0001
Freeze–thaw cycles				
Study (year)	Study design	Cycles and Temperature	Study population	Results
Bao et al.^41^	Effect of freeze–thaw on albumin and creatinine in urine samples	5 cycles (freeze: −30 °C, thaw: room temperature)	Patients with chronic kidney disease (*n* = 53)	Albumin and creatinine remained stable
Zhang et al. (2015)^42^	Effect of freeze–thaw on total protein, albumin, and calcium in urine samples	6 cycles (freeze: −20 and −80 °C, thaw: room temperature)	Patients with chronic kidney disease (*n* = 11)	All biomarkers freezed at −20 and −80 °C significantly decreased versus baseline: Total protein: - Freeze -20 °C: 2.48 ± 1.51 vs 0.75 ± 0.58 g/L; P < 0.05 - Freeze -80 °C: 2.48 ± 1.51 vs 0.96 ± 1.1 g/L; P < 0.05 Albumin: - Freeze -20 °C: 1.67 ± 1 vs 0.73 ± 0.66 g/L; P < 0.05 - Freeze -80 °C: 1.67 ± 1 vs 0.59 ± 0.39 g/L; P < 0.05 Calcium: - Freeze -20 °C: 1.64 ± 1.52 vs 0.69 ± 0.6 umol/L; P < 0.05 - Freeze -80 °C: 1.64 ± 1.52 vs 0.94 ± 0.92 umol/L; P < 0.05
Holder et al.^31^	Effect of freeze–thaw on 8-iso-PGF_2α_ while developing an analytical method by mass spectrometry	3 cycles (freeze: −70 °C, thaw: room temperature)	2 urine pools from healthy subjects	8-iso-PGF_2α_ remained stable
Present study	Effect of multiple freeze–thaw on 11-dehydro-TxB_2_, 8-iso-PGF_2α_ and creatinine in frozen urine samples with and without antioxidant and in chromatographic extracts of urine	10 cycles (freeze: −80 °C, thaw: in water bath at 25 °C for 10 min)	40 urine samples with and without antioxidant and 10 chromatographic extracts	11-dehydro-TxB_2_ and creatinine remained stable in urine samples with and without antioxidant and in chromatographic extracts of urine 8-iso-PGF_2α_ remained stable in urine samples with antioxidant in chromatographic extracts of urine 8-iso-PGF_2α_ significantly increased versus baseline after cycle 6 in urine samples without antioxidant; *P* < 0.0001

°C: degrees Celsius; PG: prostaglandin; TX: thromboxane.

To compare the stability of these molecules in different suspension media, in addition to the physiological urine milieu, we re-measured the chromatographic extracts from urine, which contain the purified lipid fraction in PBS. The 11-dehydro-TxB_2_ levels were stable also in PBS milieu, while the 8-iso-PGF_2α_ levels progressively decreased starting from approximately 4 months of storage. This progressive decrease in 8-iso-PGF_2α_ may be related to its chemical structure that includes a cyclopentane^33^ which is possibly less stable in PBS at pH 7.4 than the oxane ring of 11-dehydro-TxB_2_^18,33^. Eleven-dehydro-TxB_2_ has extra chemical resonance forms due to the double-bonded oxygen which may further stabilize the molecule^33^, while the number and position of the hydroxyl groups in the 8-iso-PGF_2α_ molecule may increase its reactivity and instability^34^. Since the 8-iso-PGF_2α_ was stable in urine but not in chromatographic extracts, we can hypothesize that the molecule is less stable at higher pH, as for PBS versus urine and/or in solutions containing salts like ethylene-diamine-tetra-acetic acid. Further investigations will be needed to clarify this difference in stability.

Concerning urinary creatinine, previous studies had investigated its stability in different storage conditions and time-intervals, as summarized in Table 2. Creatinine levels were stable in urine samples kept at 37 °C for 30 days, while at > 55 °C creatinine levels decreased (Table 2)^35^, likely due to temperature-driven degradation^36^. We had previously studied urine samples kept for 7 days at room temperature and observed stable concentrations over this short time interval as well (Table 2)^24^. Creatinine was also stable in samples stored at −22 °C over 15 years (Table 2)^37^, which is consistent with our findings. Notably, we studied urine samples from different conditions (healthy, diabetes mellitus and cancer) and creatinine values were consistently stable in all groups (Table 1).

Since the same urine samples stored in biobanks are usually used multiple times, undergoing multiple freeze–thaw cycles, we investigated the effect freeze–thaw cycles on both urine and extracted samples. Freeze–thaw cycles had been reported to variously affect some urinary metabolomic profiles^38^, with acylcarnitine and hexose^39^, acetate, benzoate and succinate significantly increasing while formate and urea decreasing after 8 freeze/thaw cycles^40^. Urinary albumin appeared stable in urine samples up to 5 freeze–thaw cycles and decreased from cycle 6 (Table 2)^41,42^. Urinary creatinine was reported stable over 8 cycles^40^ which is consistent with our data. 8-iso-PGF_2α_ and 11-dehydro-TxB_2_ have been previously studied in urine and PBS medium after a maximum of 3 freeze‐thaw cycles (Table 2)^31,41,43,44^. Our data confirm and expand the stability of 11-dehydro-TxB_2_ and creatinine in urine up to 10 consecutive freeze–thaw cycles, while a significant progressive increase in 8-iso-PGF_2α_ and hydrogen peroxide concentrations were observed starting from 5 to 6 cycle in samples without antioxidant. Approximately 40% of 8-iso-PGF_2α_ has been shown to be excreted in human urine as glucuronide conjugate and increasing pH has been reported to increase glucuronidase hydrolysis and the concentration of un-conjugated F_2_ isoprostane^45^. However, our experiments showed no changes in pH in urine samples by freezing and thawing, so it is unlikely that the increase in 8-iso-PGF_2α_ results from release of unconjugated compound. Increased concentration of H_2_O_2_ triggered by multiple freezing–thawing may lead to a non-enzymatic oxidation of AA from cell membrane residues or other contaminants in urine. Interestingly, PLA_2_ has been found in urine of mice^46–48^, healthy subjects and patients^46,48,49^ and reported to be activated by freeze–thaw cycles by urinary invertase^39^. Moreover, freezing–thawing of cells and biological fluids has been reported to increase different reactive oxygen species, mostly H_2_O_2_ and superoxide anion^50–52^. Consistent with this hypothesis, in the chromatographic extracts eluted in clean PBS with no contaminants, freeze–thaw cycles did not affect the 8-iso-PGF_2α_. Whichever the origin of free AA in urine, our data indicate that it may undergo oxidation into 8-iso-PGF_2α_ as indicated by the increased concentration of H_2_O_2_that reflects the oxidation level in the sample^28^. Consistently, 8-iso-PGF_2α_ was stable when the antioxidant 4-hydroxy-TEMPO was added, which also blocked H_2_O_2_ increase.

Our study has some limitations: we did not investigate the stability of those biomarkers in urine samples and chromatographic extracts under different storage temperatures. However, since these metabolites were stable at −40 °C, it can be assumed that −80 °C storage would give similar results, while the stability at −20 °C may be shorter. Previous studies have reported the stability of 8-iso-PGF_2α_ at −70 °C for up to 2 years^31^. Furthermore, we have not studied the stability of 8-iso-PGF_2α_ during freeze–thaw cycles in urine samples with PLA_2_ inhibitors, nor measured AA levels in urine samples. Also, in the freeze–thaw experiments small urine aliquots underwent rapid freezing at −80 °C but freezing at −40 °C or higher was not tested.

In conclusion, we showed that urinary 11-dehydro-TxB_2_, 8-iso-PGF_2α_ and creatinine are stable in urine samples stored for a decade at −40 °C. Urinary 8-iso-PGF_2α_ levels were stable for shorter time in chromatographic extracts and increased by multiple freeze–thaw cycles in urine. These data could inform correlative science projects associated with large clinical datasets analysing samples stored in biobanks for several years and undergoing multiple cycles of freezing and thawing.

## Data Availability

The dataset analyzed in the current study can be acquired from the corresponding author upon motivated request.
